# Biostimulation proved to be the most efficient method in the comparison of in situ soil remediation treatments after a simulated oil spill accident

**DOI:** 10.1007/s11356-016-7606-0

**Published:** 2016-09-27

**Authors:** Suvi Simpanen, Mari Dahl, Magdalena Gerlach, Anu Mikkonen, Vuokko Malk, Juha Mikola, Martin Romantschuk

**Affiliations:** 1 Department of Environmental Sciences, University of Helsinki, Niemenkatu 73, 15140 Lahti, Finland; 2 Department of Biological and Environmental Science, University of Jyväskylä, Survontie 9 C, 40014 Jyväskylä, Finland; 3 Mikkeli University of Applied Sciences, Patteristonkatu 3, 50100 Mikkeli, Finland

**Keywords:** Hydrocarbon contamination, Soil bioremediation, Biodegradation, Biostimulation, Chemical oxidation, Molecular monitoring

## Abstract

The use of in situ techniques in soil remediation is still rare in Finland and most other European countries due to the uncertainty of the effectiveness of the techniques especially in cold regions and also due to their potential side effects on the environment. In this study, we compared the biostimulation, chemical oxidation, and natural attenuation treatments in natural conditions and pilot scale during a 16-month experiment. A real fuel spill accident was used as a model for experiment setup and soil contamination. We found that biostimulation significantly decreased the contaminant leachate into the water, including also the non-aqueous phase liquid (NAPL). The total NAPL leachate was 19 % lower in the biostimulation treatment that in the untreated soil and 34 % lower in the biostimulation than oxidation treatment. Soil bacterial growth and community changes were first observed due to the increased carbon content via oil amendment and later due to the enhanced nutrient content via biostimulation. Overall, the most effective treatment for fresh contaminated soil was biostimulation, which enhanced the biodegradation of easily available oil in the mobile phase and consequently reduced contaminant leakage through the soil. The chemical oxidation did not enhance soil cleanup and resulted in the mobilization of contaminants. Our results suggest that biostimulation can decrease or even prevent oil migration in recently contaminated areas and can thus be considered as a potentially safe in situ treatment also in groundwater areas.

**Electronic supplementary material:**

The online version of this article (doi:10.1007/s11356-016-7606-0) contains supplementary material, which is available to authorized users.

## Introduction

Oil spill accidents are a widespread problem due to the vast production, refining, storage, and distribution of petroleum-derived products. Terrestrial oil spillages are more common than aquatic ones, although they typically pollute areas only locally and receive less publicity than the dramatic marine tanker or platform incidents (Ivshina et al. 2015). According to the data collection organized by the European Soil Data Centre in 2011–2012, mineral oil contributes to 24 % of soil and 22 % of groundwater contamination in Europe while benzene, toluene, ethylbenzene, and xylene (BTEX) contributions are 10 and 15 %, respectively (van Liedekerke et al. 2014; Panagos et al. 2013). Mineral oil contamination is common especially in Belgium, Hungary, and Lithuania, where 50 % or more of the solid matrix contamination are affected by oil, while in Finland, the proportion is 39 % (van Liedekerke et al. 2014). Transport spills on land, including oil spill sites and other hazardous substance spill sites, have caused 7.9 % of the local soil contaminations in Europe and 10.8 % of soil contaminations in Finland (van Liedekerke et al. 2014; Panagos et al. 2013). Approximately 10 million tons of hazardous substances are transported annually on the Finnish roadwork, of which 80 % are flammable liquids, mainly oil products, and the annual number of accidents is around 150 (Kallio and Mäkelä 2012).

The most important action in the incident of a terrestrial oil spill is to prevent the contamination of groundwater and surface waters. The techniques to control and minimize the oil spill spread in terrestrial areas include (i) soil berms, sorbent barriers or trenches which can enclose the soil and prevent oil flow into the soil, (ii) slurry walls constructed into a vertically excavated trench to enclose or redirect the contaminated groundwater, and (iii) viscous liquid barriers injected in the subsurface to isolate the contaminants (Ivshina et al. 2015). The extent of contamination also depends on how fast the post-spill actions are started. On the land, the prediction of movement and effects of spill are more accurate than in aquatic environments and the response or remediation strategies are thus easier to design. The slow weathering processes in soil and the strict standards usually set for the soil cleanup are other factors that help in removing most of the spilled oil (Ivshina et al. 2015).

Conventional and still often used remediation techniques are based on soil excavation, transportation, and off-site treatment. The data provided by 13 European countries revealed that in most countries, the ex situ-based techniques are used in over 70 % of the contaminated sites, with excavation and disposal applied in about 30 % of the sites, while the in situ-based treatments are rare (van Liedekerke et al. 2014). The exceptions are Malta and Netherlands, where in situ remediation techniques were mostly applied for contaminated soils. In Finland, the proportion of in situ treatments was 10 %, consisting mainly of in situ physical and chemical treatments (van Liedekerke et al. 2014). According to the US Environmental Protection Agency’s (EPA) Superfund program (responsible for the cleanup of the nation’s most contaminated land), in situ treatment was used in half of the documented cases during years 2009–2011, the most frequent techniques being soil vapor extraction, chemical treatment, and solidification/stabilization. The most common ex situ techniques were physical separation and solidification/stabilization (US Environmental Protection Agency 2013). Ex situ treatments are easier to control and monitor, but they usually cause high costs, health risks, waste production, and ecosystem disturbance at the excavated site, and consequently, the use of in situ methods has been suggested as one potential solution to enhance the eco-efficiency of contaminant land management (Sorvari et al. 2009). The low use of in situ methods is often explained by doubts regarding their efficiency and risk reduction, long-term ecological effects, time consumption, and lack of data on their suitability especially in Nordic conditions (Sorvari et al. 2009). Nevertheless, the development of remediation technologies has increasingly started to focus on in situ treatments, where biological and chemical pathways are used to enhance the subsurface decomposition of the contaminant (Sutton et al. 2011).

Micro-organisms have a natural capacity to transform or mineralize naturally occurring organic molecules, and this can be utilized in the in situ remediation of oil-contaminated areas. Also, anthropogenic chemicals such as the fuel oxygenates methyl tertiary butyl ether (MTBE) and ethyl tertiary butyl ether (ETBE), used as octane enhancers in gasoline, can be biodegraded by some microbes (Lopes Ferreira et al. 2006). Monitored natural attenuation, where the contaminant decrease without human input is regularly determined, has been used as an acceptable remediation strategy in many countries (Vogt and Richnow 2014). Although biodegradation is considered the main mechanism for contaminant removal in this strategy, the physical and chemical processes such as evaporation, dispersion, sorption, and dilution can also contribute to the contaminant reduction (Vogt and Richnow 2014). The adaptation and biodegradation of contaminants by indigenous microbes can, however, be slow if conditions do not promote microbial activity (Kauppi et al. 2011; Vallejo et al. 2001). To enhance the natural attenuation, microbial activity can be stimulated by optimizing the environmental conditions such as nutrient content, oxygen or other electron acceptor accessibility, pH, temperature, and redox conditions (Vogt and Richnow 2014; Kauppi et al. 2011; Delille et al. 2007; Margesin 2000).

While the biostimulation-based methods are generally slow, the in situ chemical oxidation (ISCO), by which organic contaminants can be destroyed or converted into more biodegradable forms, is considered fast (Sutton et al. 2011). The ISCO techniques have mostly been applied and developed in the USA, whereas in Europe, technological and regulatory constraints have limited their use (Baciocchi 2013). The ISCO treatment is implemented by distributing a chemical oxidant into the subsurface, the most commonly used reagents being hydrogen peroxide (H_2_O_2_), permanganate, persulfate, and ozone (Baciocchi 2013; Sutton et al. 2011). The use of hydrogen peroxide is based on the Fenton’s reaction, where the reaction between H_2_O_2_ and ferrous iron produces hydroxyl and other radicals (ITRC 2005). The classic Fenton’s process is applied using low H_2_O_2_ concentrations, ferrous iron as a catalyst, and a pH of 2–4, while the applied modifications (modified Fenton’s process) include high concentrations of H_2_O_2_ or calcium peroxide, neutral pH, and chelating agents for iron solubilization or H_2_O_2_ stabilization (Baciocchi 2013; ITRC 2005). The iron minerals of the soil can also act as a catalyst in H_2_O_2_ oxidation (Kong et al. 1998), in which case, the slower reaction rates might allow deeper infiltration of hydroxyl radicals (Ferguson et al. 2004). In their studies, Goi et al. (2006, 2009) found that a Fenton-like treatment, where H_2_O_2_ was added at natural soil pH and iron content, could reduce the oil contaminant concentrations but less than when the pH was lowered. However, as an in situ application, the Fenton-like treatment at soil natural pH was found to be technologically more feasible, more cost-efficient, and less harmful on soil microbial community, thus allowing subsequent biodegradation of residual contaminants (Goi et al. 2006). The combination of chemical and biological treatments has similarly been found to be an effective remediation method (Sutton et al. 2011; Goi et al. 2006).

The remediation of hydrocarbon-contaminated soil using bioremediation, chemical oxidation, or the combination of these two methods has been studied in numerous laboratory and field experiments. However, comparisons of these methods in large-scale, replicated experiments and natural conditions are lacking. In addition, in most experiments, the focus has only been on the decrease of contaminants in soil, not on the potential side effects of these practices in the field conditions, such as infiltration of contaminants or reagents into the groundwater. In this experiment, we simulated a real tank truck accident taken place in Iisalmi, Finland, where the accident resulted in a fuel spill of diesel and gasoline into the groundwater area (Centre for Economic Development, Transport, and the Environment North Savo 2013). Because of the groundwater formation, these areas are the worst possible places for accidental oil spill. We downscaled the fuel spill effects in controlled conditions at the Jokimaa Soil Research Centre, where 2-m deep soil columns were built to mimic the sandy and gravelly ridge area at the real accident site. Using contaminated soil, we compared three in situ remediation methods, i.e., natural attenuation, biostimulation, and chemical oxidation, all in the prevailing climatic conditions. During the 16-month study, contaminant concentrations in soil and water passed through the soil were analyzed and the effects of oil and remediation treatments on bacterial abundance and community profiles were monitored. Our objectives were (a) to evaluate the effectiveness of cleaning treatments on oil-contaminated soil in boreal conditions and (b) to evaluate the risks that these practices might cause via mobilization of contaminants and other harmful compounds generated during the remediation process.

## Materials and methods

### Experimental setup

**Fig. 1:**
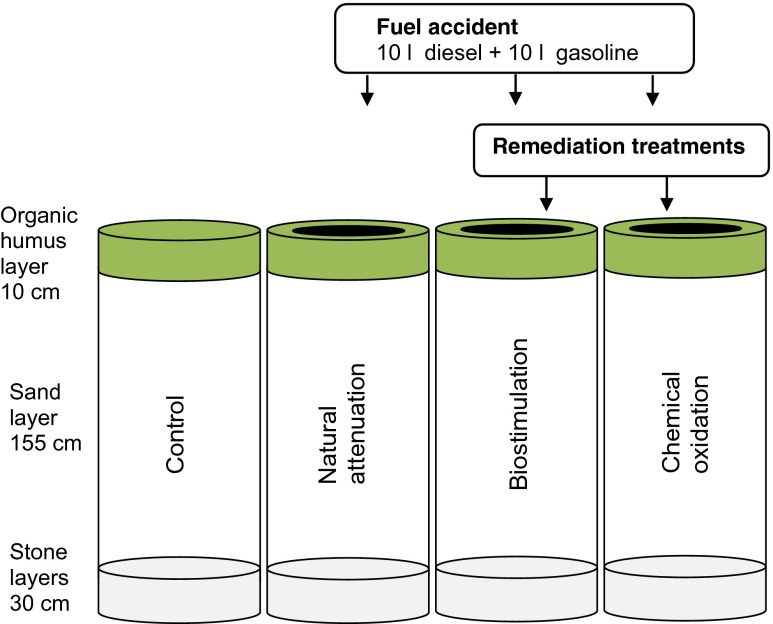
Graphic presentation of the experiment setup. The height of the lysimeters was 195 cm and volume 1.7 m^3^. Each treatment was prepared in duplicate (total of eight lysimeters)

The experiment was performed at the Soil Research Center in Lahti, Finland, where eight steel cylinders or lysimeters (volume 1.65 m^3^, diameter 1.04 m, height 1.95 m) were embedded into the soil and exposed to seasonal variation. The soil simulating a ridge area was constructed by using a 10-cm layer of stone (size 30–100 mm), a 20-cm layer of crushed stone (3–6 mm), a 155-cm layer of sand (0.1–4 mm), and a 10-cm layer of organic humus (Fig. 1). The sand layer was packed by saturating it with water for 30 min, draining the excess water, and leaving the sand to stand for 1 month. The organic humus layer was cut out as two large pieces from a pine forest in Hollola, Finland (61° 00′ 15.0″ N, 25° 29′ 10.4″ E), in the beginning of May 2012 and set on the top of the sand layer. The setup was then let to stand for another month. Among the completed lysimeters, the combined weight of stone and sand layers varied between 2412 and 2723 kg and the weight of the humus layer between 43 and 69 kg.

Six of the lysimeters were contaminated in the beginning of June 2012 (day 0). A fuel mixture containing 10 L Neste Green diesel and 10 L Neste gasoline 95E 10 (supplier Neste Oil Oyj 2012), a combination of fuels in the Iisalmi accident, was spread on the top of the humus layer in each lysimeter in 1.5 min (Fig. 1). The spill volume was scaled so that the soil layer could retain the spill and no overflow through the soil happened right after fuel adding (calculative fuel concentrations in soil were similar with those measured in the accident site 2 years after the spill). Two lysimeters were left uncontaminated and served as controls. After contamination, the soils were left to settle for 4 months to allow the contamination to spread evenly over the soil column and to reach deeper soil layers.

### Remediation treatments

Each of the three in situ remediation methods, biostimulation, chemical oxidation, and natural attenuation, were applied in two lysimeters (Fig. 1). Remediation treatments were started 4 months after the simulated spill (day 138). This mimicked also the situation at the actual site of the accident, where further treatment of deeper layers was planned 2 years after the accident. The most contaminated organic humus layer was first removed, and the remediation treatments were implemented on the sand layer by pouring 10 L of treatment liquids over the soil twice a month during the entire unfrozen period (11 additions in total).

The biostimulation treatment consisted of nitrogen added as urea (Yara Suomi Oy, Finland) dissolved in 5 mM K_2_HPO_4_/NaH_2_PO_4_ buffer (pH 7). The concentration of urea-N was initially 8.2 g N L^−1^ but was decreased to 7.5 g N L^−1^ in the spring 2013 when the hydrocarbon concentrations in the sand had decreased. The total carbon content of the sand was calculated using the soil organic matter content and showed that the C:N ratio was approximately 100:1 after adding the urea. Urea was not added in every treatment liquid, only when the ammonium and nitrate concentrations measured in the water passing through the soil were not detectable. The oxygen concentration in the treatment liquid was increased before pouring by pumping air through air stones for 1 h using vacuum pump (Piston Pump WOB-L 2534, Welch-Ilmvac, Germany) with a flow rate of 28 L min^−1^. The chemical oxidation treatment was implemented as a modified Fenton’s reaction, where 10 % (*w*/*w*) H_2_O_2_ solution (INTEROX® AG Bath 35 % Hydrogen Peroxide, Solvay Chemicals International) was used without soil acidification or addition of soluble iron catalyst. The natural attenuation treatment was left without any amendments to illustrate the self-remediating properties of the soil.

### Sampling

At the start of the experiment (sampling time 0 day), samples were taken horizontally from the middle of the sand layer at the depth of 85 cm using openings on the sides of the lysimeters. However, this turned out to be an unsuitable technique, which did not give representative samples, and all later samples were collected vertically using an auger (diameter 5 cm) and were divided into the following two layers: 0–50 and 50–100 cm. Two replicate samples were taken from each lysimeter, and the sampling was repeated six times, i.e., at day 0 before the contamination; at days 82 and 126 after the contamination before the treatments; and at days 333, 391, and 466 after the contamination and the treatments. At the end of the experiment (467–469 days after the contamination), the lysimeters were emptied and additional samples were collected from the depths of 0–75 and 75–150 cm. From both depths, altogether 55 samples (50 g each) were collected throughout the whole sand layer and combined. These combined samples were studied as three analytical (pseudo) replicates to quantify the analytical reproducibility. Soil samples were stored at −70 °C for molecular analysis and at −20 °C for other analyses.

The water that passed through the soil (so-called “center water”) and the water passing along the lysimeter wall (so-called “side water”) were collected and measured separately. Samples of 0.5 L were taken from the center water that was collected into 10-L glass bottles located under each lysimeter, and the side water was used for measuring volume only. The side water volume was 2–12 % of the total water volume that passed through the soil, except in one lysimeter of chemical oxidation treatment, where the amount was 32 %. Water samples were collected 4 and 13 days after the soil contamination in all lysimeters and after that whenever a glass bottle got full (one to eight times per month; the sampling frequency varied among the lysimeters after the start of the remediation treatments). For the chemical analysis, 100-mL water samples were stored at −20 °C from each sampling and later combined to estimate the water contaminants for different periods of the experiment. The contaminant concentrations were measured from the center water, but the contaminant amounts in water were calculated using total water volumes. For other analyses, water samples were stored at 4 °C. In the contaminated lysimeters, the fuel mixture migrated partly through the soil as a non-aqueous phase liquid (NAPL), forming an immiscible phase on the surface of the water in the collecting bottle. The NAPL phase was removed by pipetting before water sampling, and the volume of NAPL was measured.

The bacterial 16S ribosomal ribonucleic acid (rRNA) gene density of the leachate water was analyzed when the originally clear color of the biostimulation water changed to dark brownish (in other treatments, the color remained clear). Analysis was performed using samples taken from center bottles, and in contrary to the chemical analysis, samples from different dates were not combined.

### Chemical and physical analyses

Volatile organic compounds (VOCs), including the total C_5_–C_10_ fraction, BTEX compounds (benzene, toluene, ethylbenzene, and m + p xylenes), and oxygenates (MTBE, TAME, TAEE, ETBE), were analyzed according to standards ISO22155 (soil) and ISO114223–1 (water). Methanol extraction was used for soil, and determination was done with a static headspace technique using gas chromatography/mass spectrometry. Total C_10_–C_21_ and C_22_–C_40_ fractions were analyzed according to standards ISO16703 (soil) and ISO9377–2 (water). Determination was done using hexane-acetone extraction (soil) or hexane extraction (water), florisil purification, and gas chromatography/flame ionization detector. All hydrocarbon content analyses were performed at SGS Inspection Services Ltd. in Kotka, Finland.

Soil dry mass was determined using 2–6-g subsamples, dried overnight at 105 °C, and the organic matter content was measured as loss on ignition (4 h in 550 °C) (Kauppi et al. 2011). For pH measurements, 10 g of soil was shaken with 50 mL 0.01 M CaCl_2_ for 30 min (250 rpm) and incubated at room temperature for 12 h. Nitrogen was measured from water samples semi-quantitatively using ammonium and nitrate test strips (Merckoquant, Germany).

### Molecular analyses

#### DNA extraction

Total DNA was extracted from 1 g of soils and 10 mL of water samples using PowerSoil® DNA Isolation Kit (MoBio Laboratories Inc., Carlsbad, USA) according to the manufacturer’s instructions with the exceptions of centrifugation being always 1 min at 11,000×*g*. The DNA yield was confirmed by agarose gel electrophoresis (1.5 % agarose gel run at 150 V for 1 h) and visualized with ethidium bromide. The extracted DNA was stored at −70 °C.

#### Quantitative PCR

To quantify the total bacterial communities in samples, bacterial 16S rRNA genes were amplified and monitored using Light Cycler® 96 Instrument (Roche Diagnostics, Germany). In all quantitative PCR (qPCR) reactions, the total reaction volume was 20 μL and reactions were carried out using Fast Start Essential DNA Green Master Kit (Roche Diagnostics). The primers for total bacterial 16S rRNA gene quantification were pE and pF (Ekman et al. 2007; Kanto Öqvist et al. 2008). In each reaction, the mixture contained 2 μL undiluted or 1:10–1:1000 diluted DNA, 1× premix, 0.1 μM each primer (Oligomer), and 0.2 mg mL^−1^ bovine serum album (BSA; Thermo Scientific). The temperature program was 10 min at 95 °C, followed by 30 cycles of 10 s at 95 °C, 20 s at 57 °C, and 30 s at 72 °C. To check the specificity of amplification, the melting curve was determined after each temperature program as follows: 10 s at 95 °C, 60 s at 65 °C, 1 s at 97 °C, and 30 s at 37 °C.

Positive control was used to create a five-point calibration curve by tenfold serial dilution. Positive control was DNA from a pure culture of bacterium *Cupriavidus necator* JMP134 (DSM 4058) (DSMZ, Germany), and sterile water was used as a negative control. The amplification efficiency for standards was 80–101 %.

#### Length heterogeneity PCR

Bacterial community structures were monitored by amplifying the hypervariable regions V1–V3 of the 16S rRNA gene with the general bacterial primers fD1 and 5′FAM-labeled PRUN518r (Mikkonen et al. 2011). The volume of one PCR reaction was 50 μL, containing 2 μL undiluted DNA extract, 0.2 mM of each dNTP (Thermo Scientific), 0.3 μM of each primer (Oligomer), 0.7 mg mL^−1^ BSA (Thermo Scientific), 1× PCR buffer (Biotools), and 1 U DNA polymerase (Biotools). The amplification was done in a DNA Engine DYAD^™^ thermal cycler (MJ Research Inc., USA) with the following program: 5 min at 95 °C followed by 28 or 30 cycles of 45 s at 94 °C, 1 min at 55 °C, 1 min at 72 °C, and finally 5 min at 72 °C. The PCR products were checked by agarose gel electrophoresis (1.5 % agarose gel run at 100 V for 1 h). Fragment analysis by polyacrylamide capillary electrophoresis (ABI 3130 XL, Applied Biosystems) was outsourced to DNA Sequencing and Genomics Laboratory Core Facility, University of Helsinki, Finland, using GeneScan 600 LIZ (Applied Biosystems) as sizing standard.

### Community fingerprint data analysis and statistical analysis

Data analyses were performed using BioNumerics 6.0 (Applied Maths, Belgium), SPSS 15.0 statistical package (SPSS Inc., Cary, NC, USA), and freely downloadable FORTRAN program CAP15 (canonical analysis of principal coordinates; Anderson 2002).

The length heterogeneity PCR (LH-PCR) fingerprint data was processed and analyzed as described by Mikkonen et al. (2011). The signal strengths of fingerprint electropherograms were checked using Peak Scanner^™^ Software v1.0 (Applied Biosystems) and the normalization (alignment) of the fingerprints with standards using BioNumerics 6.0. The fingerprint active area was restricted to amplicon size 460–560 bp, which corresponded to 1252 data points, and for comparative visualization, the aligned fingerprints were normalized by total fluorescence.

The pairwise LH-PCR community profile similarities were calculated as Pearson’s correlation coefficients in BioNumerics 6.0 allowing 1-bp optimization. The community similarity values were Fisher transformed to normalize the skewed distribution before the calculations of mean, standard deviation (SD), and 95 % confidence intervals. The effects of contamination and remediation treatments (contaminated and treated soil versus uncontaminated control soil) and the effects of bare remediation treatments (contaminated treated soil versus contaminated untreated soil) were calculated as sampling day average similarities between treatments. The effects of the treatments on microbial community were calculated as dissimilarity values, by subtracting the Pearson’s correlation similarity values from 1. The significance of the effects was evaluated by permutation test in the CAP program (generalized discriminant analysis) with 9999 permutations.

To compare the treatment effects on bacterial community diversity, the Shannon diversity index *H*′ was calculated from the fingerprint curve data, normalized by the total fluorescence intensity (Mikkonen et al. 2011). Mean and SD of *H*′ were calculated from the four replicates without transformation. Non-parametric Kruskall-Wallis test was used to test if Shannon *H*′ differed in the different treatments.

The statistical significance of the treatment effects on soil contaminant concentrations and microbial amounts was analyzed using nested one-way ANOVA models, where the lysimeter was nested within the treatment (two samples for each lysimeter, two replicates for each treatment). For the water variables, which had only one sample for each lysimeter, the data were analyzed using non-nested one-way ANOVA followed by Student-Newman-Keuls post hoc test. Both in the soil and in the water data, the control treatment was included only in the analysis of the bacterial data. The homogeneity of variances was evaluated visually, and if necessary, log or square root transformations were used to meet the requirements of the analysis.

## Results

### Contaminant contents

**Fig. 2:**
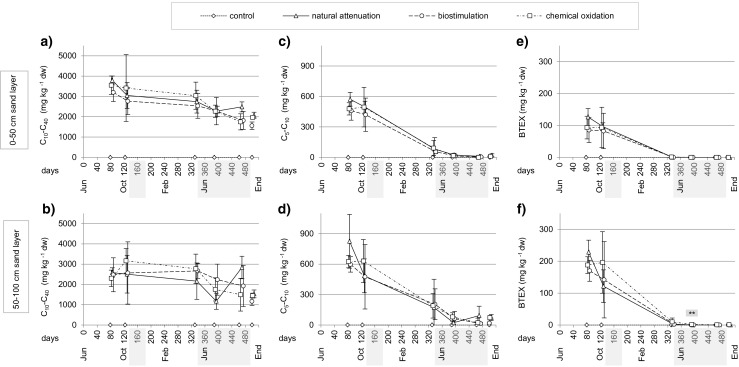
Concentrations of **a**, **b** oil hydrocarbon C_10_–C_40_, **c**, **d** oil hydrocarbon C_5_–C_10_, and **e**, **f** BTEX compounds (mean ± SD) in 0–50- and 50–100-cm sand layers (end samples in 0–75- and 75–150-cm sand layers). The *gray bars* in *x* axis indicate the periods when treatment solutions were added into the soil. Statistically significant differences among the treatments are indicated with an *asterisk* above each data point (*P < 0.05, **P < 0.01, ***P < 0.001)

Four days after contamination, the average contaminant concentrations in the top humus layer were 35,100 ± 16,100 mg C_10_–C_40_ hydrocarbons; 10,300 ± 4600 mg C_5_–C_10_ hydrocarbons; 6200 ± 3300 mg BTEX compounds; and 800 ± 500 mg TAEE kg^−1^ dry weight (dw) humus, whereas in the sand, the concentrations were 100 or even less of those in the humus. At day 82, oil hydrocarbon C_10_–C_40_ concentrations were higher in the upper 0–50-cm sand layer, whereas concentrations of oil hydrocarbons C_5_–C_10_ and BTEX compounds were higher in the lower 50–100-cm layer, indicating that the smaller compounds migrate faster in the soil and vaporize near the surface (Fig. 2). The reduction of latter groups was also clear at the end of the experiment when C_5_–C_10_ concentrations had decreased by 89–99 % and BTEX concentrations by 99–100 % in all treatments. The decrease in the concentration of oil hydrocarbons C_10_–C_40_ in soil was lower, average reduction being 45–51 % in the upper sand layer and 37–53 % in the lower sand layer (calculated using the end sampling results). The remediation treatments differed statistically significantly only in BTEX concentrations of the lower sand on day 391 (Fig. 2), in which case the chemical oxidation treatment, having the highest concentrations, differed from other treatments. From the four gasoline oxygenates analyzed from the sand, only TAEE was observed at day 82 (maximum 1.8 mg kg^−1^ dw and 4.7 mg kg^−1^ dw in the upper and lower sand layers, respectively).

**Fig. 3:**
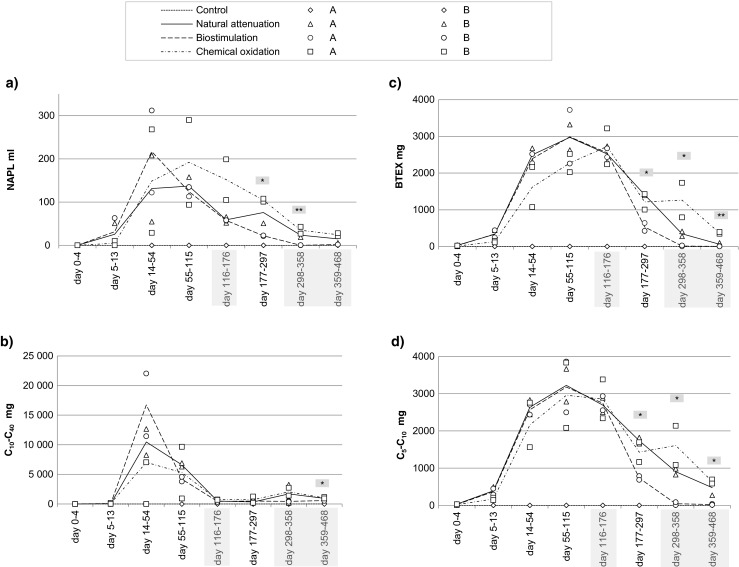
Volume of NAPL and mass of oil hydrocarbons C_10_–C_40_, C_5_–C_10_, and BTEX compounds in the water passed through the soil during each period. The *line* represents the average value, and the *markers* show the values of the two replicate lysimeters. The *gray bars* in *x* axis and the statistically significant differences are the same as in Fig. 1

Low concentrations of C_5_–C_10_ (3–9 mg L^−1^), BTEX (2–8 mg L^−1^), ETBE (4–14 mg L^−1^), and TAEE (6–17 mg L^−1^) were observed in the water that had passed through the soil 4 days after contamination, while C_10_–C_40_ (0.04–16 mg L^−1^) were not observed until 13 days after contamination. The NAPL phase was measurable for the first time on day 13 in water samples of three lysimeters and by the day 25 in water samples of all lysimeters. In general, over half (52–95 % and in the case of ETBE 100 %) of the total quantity of the contaminant that ended up into the water during the experiment migrated there before the remediation treatments were started (Fig. 3 and Table S1 in the Supplementary Data online). The exception was the chemical oxidation treatment, where NAPL, C_5_–C_10_, and BTEX quantities in the leachate during the first 115 days were 46, 44, and 42 %, respectively, of the total leachate quantities. In the natural attenuation treatment, the total leachate into the water during the experiment was 468 ± 238 mL NAPL (mean ± SD), 21 ± 6 g C_10_–C_40_ hydrocarbons, 12 ± 1 g C_5_–C_10_ hydrocarbons, 10 ± 1 g BTEX compounds, 10 ± 2 g TAEE, and 4 ± 0.6 g ETBE.

Once the remediation treatments started, the migration of contaminants into the water decreased, and especially in the biostimulation treatment, where the reduction with respect to other treatments was statistically significant on the period of 177–358 days in NAPL, BTEX, and C_5_–C_10_ and on the period of 359–468 days in hydrocarbon C_10_–C_40_ and C_5_–C_10_ (Fig. 3). During the last period (359–468 days), leachate quantities of all contaminants were highest in the chemical oxidation treatment, and for BTEX compounds, this difference was statistically significant.

### Bacterial 16S rRNA gene abundance

**Fig. 4:**
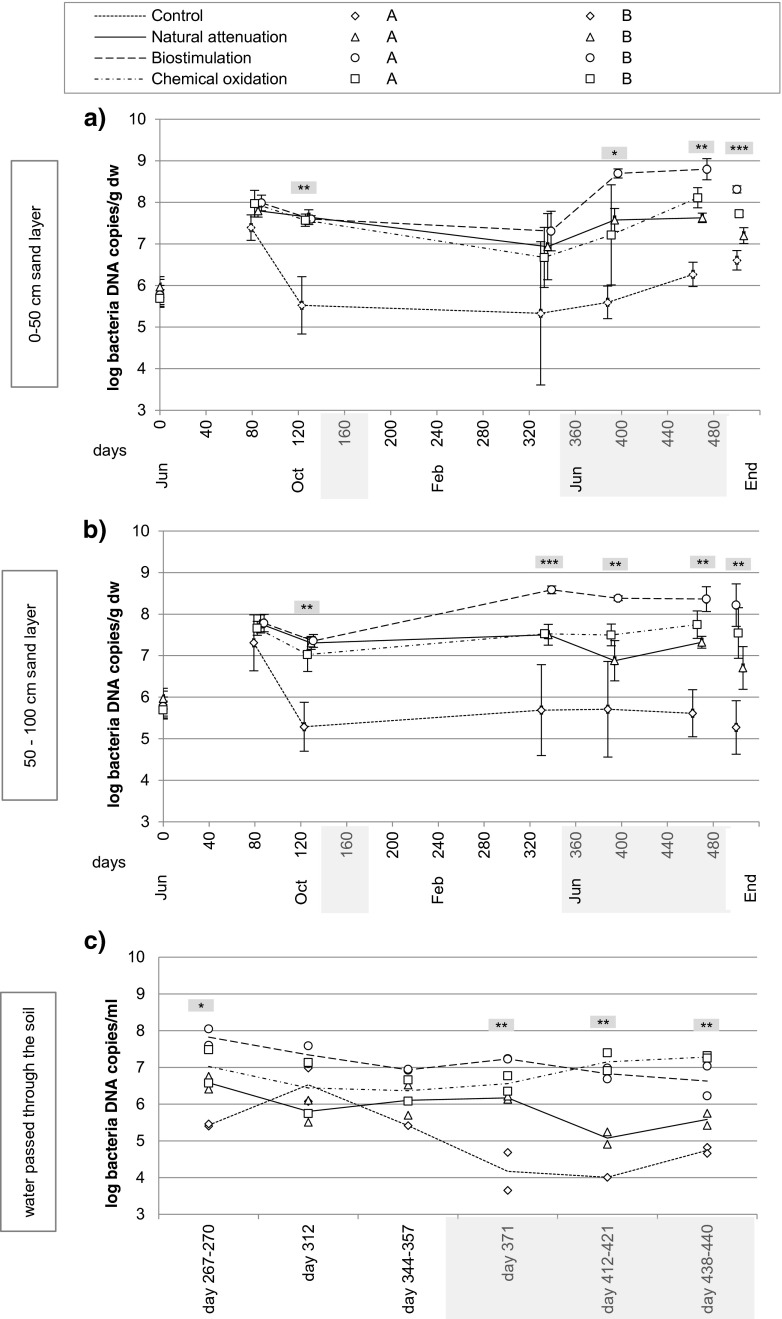
Bacterial 16S rRNA gene abundance (mean ± SD) in **a** 0–50-cm sand layer (end sample 0–75 cm) and **b** 50–100-cm sand layer (end sample 75–150 cm) and **c** in water passed through the soil (the average value as *line* and replicate values as *markers*) analyzed from samples taken on those days the bottles were emptied (water sampling days varied between lysimeters). The *gray bars* in *x* axis and the statistically significant differences are the same as in Fig. 1. Sand samples on day 0 were taken horizontally from the depth of 85 cm

The copy numbers of bacterial 16S ribosomal DNA (rDNA) were 10 to 1000 times greater in the contaminated sand than in the uncontaminated sand (Fig. 4a, b). The only exception was day 82, when the qPCR results of control sand were close to the results of the contaminated sands. Statistically significant differences were observed among the treatments on almost all sampling points during the remediation period. After the start of remediation, the increase in bacterial 16S rDNA copy numbers was greatest in the both sand layers of the biostimulation treatment, being on average 6 times greater than in the oxidation treatment and 15 times greater than in the natural attenuation treatment. In the chemical oxidation treatment, the gene abundance was on average two to three times greater than in the natural attenuation treatment.

In the leachate water, the 16S rRNA gene density was lowest in the uncontaminated control samples, and this was statistically significant on the first and fourth analyzing dates (Fig. 4c). The gene densities of the biostimulation and chemical oxidation treatment were similar especially at the end of the experiment, when the gene densities of these treatments were statistically significantly higher than those in the natural attenuation treatment and control.

### Bacterial community responses

**Fig. 5:**
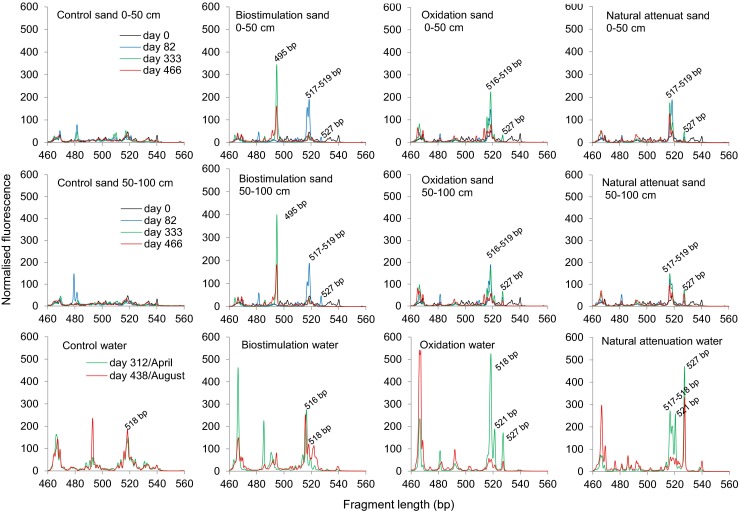
Bacterial community profiles in 0–50- and 50–100-cm sand layers and in water passed through the soil. Soil profiles are an average of two to four replicate fingerprints on days 0 (before contamination), 82 (after contamination), 333 (after the start of treatments), and 466 (at the end of experiment). Water profiles are an average of two replicate fingerprints on days 312 and 438. All fingerprints are normalized by the total fluorescence intensity. Sand samples on day 0 were taken horizontally from the depth of 85 cm

Averaged sand soil LH-PCR community profiles were calculated for days 0 (before contamination), 82 (after contamination), 333 (after the start of remediation treatments), and 466 (at the end of experiment) and water profiles for days 312 and 438. The first change in bacterial community profiles occurred after contamination, when the most dominant peak region in all contaminated sands was the amplicon length 517–519 bp, followed by the amplicon length 527 bp (Fig. 5). Some peaks from this region were visible also in water samples, including the control samples. Starting biostimulation changed soil community profiles so that the amplicon of size 495 bp appeared and the peak regions induced by contamination decreased (Fig. 5). The peak length of 516 bp was visible in some samples of the chemical oxidation and biostimulation treatments and the peak length of 521 bp in some samples of the chemical oxidation and the natural attenuation treatments. In the chemical oxidation treatment, the intensity of all distinct peaks decreased by the day 466.

**Fig. 6:**
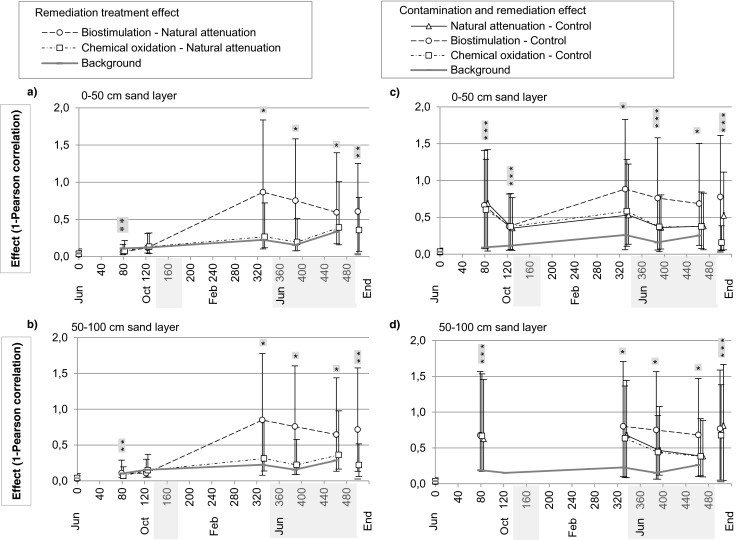
The effect of **a**, **b** remediation treatment and **c**, **d** contamination and remediation treatments on bacterial community succession in 0–50- and 50–100-cm sand layers (end samples in 0–75- and 75–150-cm sand layers) during the experiment. The effect size is shown as dissimilarity of community structures (1—curve-based Pearson’s correlation coefficient for similarity) between the contaminated remediated soil and the contaminated untreated reference (**a**, **b**) and between the contaminated remediated soil and the uncontaminated untreated reference (**c**, **d**). Mean and SD were calculated using Fisher-transformed Pearson’s correlations crossed between two to four replicates of each treatment (*n* = 8–16 for each figure point) except with the end samples, where calculations are based on five to six replicates (*n* = 30–36 for each figure point). The *asterisks* depict the significant differences (*P* < 0.05) among treatments, calculated using generalized discriminant analysis with 9999 permutations of the dissimilarity matrix. Nonsignificant “baseline dissimilarity” is shown as a *gray solid line* and is based on lower 95 % confidence limits calculated for each sampling time using an average of all within-treatment similarities (in remediation treatment effect *n* = 12–18 except for end *n* = 40–45, in contamination and remediation effect *n* = 18–24 except for end *n* = 55–60). Sand samples on day 0 were taken horizontally from the depth of 85 cm

The effects of contamination and remediation treatments on the sand soil bacterial community structure during the entire experiment were calculated as community profile dissimilarity values (1—profile Pearson’s correlation). In the contaminated sands, the biostimulation treatment caused a significant decrease in fingerprint similarity, while the effect of chemical oxidation was negligible (Fig. 6a, b). As could be expected, contamination affected microbial communities significantly on days 82 and 126, but after the start of the remediation treatments, biostimulation impact was even more pronounced, being significant from day 333 onward (Fig. 6c, d). The contamination and remediation effects were quite consistent between the upper and lower sand layers when comparing vertically taken drilled samples. However, at the end of the experiment, the treatment effects observed in drilled samples taken on day 466 were somewhat different from the effects seen in combined end samples, where the differences in bacterial community fingerprints were statistically significant among all treatments (Fig. 6a, d).

Shannon diversity index *H*′ was used to describe the changes in the bacterial profile complexity in sand soil during the experiment. Before the contamination, on day 0, the average diversity of all lysimeters was 2.9 ± 0.3 (mean ± SD). By the day 82, the diversity had decreased in all sands, but statistically significant reduction due to the contamination was seen in the upper sand layer only (Table 1). After the start of remediation, the lowest diversity was observed in the biostimulation treatment, although this effect was statistically significant only at day 391 in the lower sand layer. Chemical oxidation did not have an effect on diversity. At the end of the experiment (from day 391), the bacterial profile complexity started to recover in all treatments, being highest in the upper control sand of the last samples taken with the auger (day 466). This observation was, however, inconsistent with the upper layer end samples, where the diversity was significantly highest in the natural attenuation treatment.

**Table 1:** Changes in the bacterial community complexity in different treatments in 0–50- and 50–100-cm sand layers (end samples in 0–75- and 75–150-cm sand layers) shown as Shannon index *H*′

Depth	Treatment	82 days	126 days	333 days	391 days	466 days	End
0–50 cm	Control	2.3 ± 0.1 b	1.9 ± 0.02 a	2.1 ± 0.4 a	2.1 ± 0.03 a	2.6 ± 0.1 b	1.5 ± 0.3 a
	Natural attenuation	1.5 ± 0.6 a	1.7 ± 0.2 a	1.5 ± 0.1 a	2.0 ± 0.1 a	2.0 ± 0.3 a	2.5 ± 0.2 b
	Biostimulation	1.6 ± 0.1 a	1.6 ± 0.2 a	1.1 ± 0.4 a	1.7 ± 0.3 a	1.8 ± 0.5 a	1.4 ± 0.2 a
	Chemical oxidation	1.8 ± 0.3 a	1.9 ± 0.2 a	1.4 ± 0.3 a	1.7 ± 0.2 a	2.1 ± 0.1 a	1.7 ± 0.3 a
50–100 cm	Control	1.8 ± 1.0 a	No result	1.3 ± 1.1 a	2.4 ± 0.2 b	2.5 ± 0.2 a	2.4 ± 0.5 a
	Natural attenuation	1.9 ± 0.2 a	1.7 ± 0.2 a	1.6 ± 0.2 a	2.0 ± 0.3 ab	2.0 ± 0.2 a	2.0 ± 0.3 a
	Biostimulation	1.9 ± 0.2 a	1.7 ± 0.2 a	1.3 ± 0.3 a	1.5 ± 0.2 a	2.1 ± 0.4 a	1.9 ± 0.3 a
	Chemical oxidation	1.9 ± 0.2 a	1.8 ± 0.1 a	1.8 ± 0.1 a	2.0 ± 0.2 ab	2.2 ± 0.2 a	1.8 ± 0.2 a

The index was calculated using the normalized fingerprint profiles without traditional peak assignment. Significant differences among treatments at each sampling time are indicated by different lowercase letters and calculated using Kruskal-Wallis test (*n* = 2–4 for each treatment except for end samples *n* = 5–6). At sampling time 0 day (horizontally taken samples from the depth of 85 cm), the average Shannon index *H*′ of all lysimeters was 2.9 ± 0.3 (*n* = 16)

## Discussion

In an accidental fuel spill, the properties and amount of fuel together with the soil type, moisture content, and weather conditions determine how detrimental the accident is and how fast the fuel components reach the groundwater zone. Our pilot-scale remediation study, implemented using 2-m deep soil columns (lysimeters), was built to mimic a real tank truck accident. To get the contaminants spread evenly into the sand layer, the most contaminated humus layer was left first on the sand and the experiment was let to stand for 4 months after contamination. This also roughly reflected the situation at the actual accident site. Although there the top layer was removed within a few days after which soil was treated with soil vapor extraction to remove the most volatile fractions during the year after the accident, the deeper sand layers were still heavily contaminated 2 years after the spill (Centre for Economic Development, Transport, and the Environment, North Savo 2013). In our study, the spill volume was scaled so that the fuel concentrations in the soil represented the values that were measured at the site of the accident at the time when the further restoration was planned, i.e., after the first prevention actions and soil treatment had been performed.

The most vulnerable soils for fuel migration are the permeable sandy soils, whereas soils with lower hydraulic conductivity can delay the fuel penetration. Halmemies et al. (2003) measured the short-term (2 h) retention capacities in laboratory-scale column experiments and found that sandy till can retain 1.5–2 times and peat 3.5 times more fuel than gravely sand (measured as volume percent of retained fuel from soil volume). In the same study, the moisture content had minor effects on retention, but instead a significant effect on fuel migration velocity, the migration being faster in dry than in wet sand, as noticed also by Malk et al. (2014). When comparing migration velocities of different fuels, gasoline with low viscosity has been observed to migrate seven to nine times faster than diesel in sand (Malk et al. 2014) and three times faster in sandy till or peat (Halmemies et al. 2003). Those results are consistent with observations presented here, although we performed the spill using a mixture of gasoline and diesel. In our study, the fuel contaminants were absorbed efficiently to the top organic humus layer right after the simulated spill. Four months later, the gasoline-derived contaminants, C_5_–C_10_ hydrocarbons, BTEX compounds, and TAEE, were found in higher concentrations in the lower sand layer than the diesel-derived C_10_–C_40_ hydrocarbons, indicating faster migrating velocity of the former ones (Fig. 2). The gasoline compounds were also detected in leachate water earlier than the diesel compounds (Fig. 3).

Experimental and case studies have shown that autochthonous bacterial communities are capable of adapting to oil contamination and degradation of oil-derived compounds (Acosta-González et al. 2015; Oliveira et al. 2013; Chikere et al. 2012; Bento et al. 2005). LH-PCR has been proved to be a sensitive method to monitor the successional changes in bacterial community (Yan et al. 2016; Mikkonen et al. 2014). This was also seen in our study, where soil bacterial abundance increased (Fig. 4) and the community structure changed (Fig. 5) after the contamination. Significantly lower bacterial abundances in uncontaminated soils throughout the experiment were apparently a result of lower levels of carbon resources. Increase in bacterial growth was, however, observed also in control soils at the end of both summers (Fig. 4), indicating the effect of temperature on microbial growth. Selection for oil-degrading bacteria after the contamination reduced community complexity (Table 1) and led to dissimilar fingerprints between the control and contaminated soils (Fig. 6). In an earlier study with the same LH-PCR community analysis and sequencing of peaks in fresh oil-contaminated soil, the peak lengths 516–518 and 521–522 bp were identified to represent various genera of *Proteobacteria* and *Firmicutes* (Mikkonen et al. 2011). In the same study, the peak length 519–521 bp was identified as genus *Aquabacterium* (class *Betaproteobacteria)* and was found to dominate the community soon after contamination, a sign of being an important degrader of readily utilizable oil hydrocarbons. Amplicon lengths that were found both by us and Mikkonen et al. (2014) were the size 517 bp that was identified as *Flavobacteriacea* (*Bacteroidetes*) and *Ruminococcaceae* (*Firmicutes*) and the size 519 bp identified as *Gammaproteobacteria*. Presumably, some of the bacteria mentioned above were involved in the degradation process in our experiment.

Typical factors that limit natural soil degradation capacity are the lack of nutrients (especially N), soil anoxia, low temperature, insufficient soil moisture, or unsuitable pH (Simpanen et al. 2016; Kauppi et al. 2011; Suni et al. 2007; Vallejo et al. 2001; Margesin 2000; Romantschuk et al. 2000). When the soil C:N ratio was balanced by N amendments in the present study, bacterial growth increased but also the community structure changed. A new bacterial group, seen in LH-PCR profile as peak length 495 bp, responded to biostimulation, replaced the groups promoted by the contamination, and dominated the community until the end of the experiment (Fig. 5). In the study by Mikkonen et al. (2014), similar amplicon length in oil-contaminated and urea-fertilized soil was formed by *Actinobacteria*. N addition is clearly crucial to enhance the biodegradation, but the N type and dosage also matter; too intense fertilization has detrimental effects on microbial processes (Akbari and Ghoshal 2014; Kauppi et al. 2011; Chaillan et al. 2006; Peltola et al. 2006) and can lead to groundwater pollution. Peltola et al. (2006) observed that high urea load (C_mineral oil_:N_fertilizer_ ratio 100:19) inhibited nitrification due to the increase of soil ammonium concentration and pH, while the same load of methylene urea was well tolerated because of a slower release of N and lack of pH effect. In our study, the urea load did not increase the soil pH, but at the end of the experiment, the nitrate concentration of leaching water was 100–500 mg L^−1^, exceeding the value 50 mg L^−1^ set as a quality standard for groundwater in the European Union (Directive 2006/118/EC). Careful monitoring of N levels is therefore needed when urea is used as a N source.

Remediating soil using hydrogen peroxide has been found to be a rapid method for oil contaminant reduction in several studies (Goi et al. 2009; Villa et al. 2008; Vitolins et al. 2003), but contrasting observations have also been made (Sutton et al. 2014; Ferguson et al. 2004). As a strong oxidant, H_2_O_2_ can result in long-lasting, damaging effects on the microbial community (Ferguson et al. 2004) and can also reduce the bacterial diversity (Silva-Castro et al. 2013). In our study, chemical oxidation did not enhance contaminant degradation, and contrary to our expectations, did not destroy soil micro-biota, but instead slightly increased bacterial abundances at the end of the experiment (Fig. 4). This was probably due to the improved aerobic conditions in soil that followed peroxide degradation as observed in other studies (Sutton et al. 2011; Tsai et al. 2009; Kulik et al. 2006; Palmroth et al. 2006). The most probable reason for the ineffective oxidation in the current study was the small amount of H_2_O_2_ in relation to the oil amount. In the study by Goi et al. (2009), the removal of diesel in sand was enhanced by increasing the H_2_O_2_:diesel (*w*:*w*) ratio, the removal being over 90 % with the ratio 5:1 and over 50 % with the ratio 0.33:1. In our study, the H_2_O_2_:diesel ratio was 0.12:1 every time when peroxide was added, and most likely, a more efficient oxidation would have required higher liquid doses and infiltration of the liquid directly into the deeper layers. However, our procedure used realistic doses that would be feasible also in the field. Fenton process has earlier been observed to dissolute soil metals due to a decrease of soil pH (Villa et al. 2008), and this was also observed in our study; as soil pH decreased to 5–6 in the chemical oxidation treatment, more aluminum leached into the water, the maximum concentrations being 8.6 mg L^−1^ (in other treatments, 0.01–0.1 mg L^−1^).

During the remediation process, the treatments did not differ significantly in soil oil concentrations and the positive effect of biostimulation emerged only at the end of the experiment when the lysimeters were emptied; then, the C_10_–C_40_ concentrations were 20–25 % lower and the C_5_–C_10_ concentrations 30–75 % lower in lysimeters treated with biostimulation than in lysimeters treated with other procedures (Fig. 2). In the samples collected with an auger, the result was not as clear, but we assume that the end samples better represent the final stage of the experiment because of a more comprehensive sampling. One explanation for the biostimulation effect might be that more oil was able to dissolve into the water that never became saturated with oil due to the microbial degradation. Bacterial surfactants can also make adsorbed oil more bioavailable for degradation (Ławniczak et al. 2013; Singh et al. 2007).

Significantly less contaminants leached into the water in the biostimulation than other treatments, and when comparing the treated and untreated soils, the effect of remediation was clear (Fig. 3). Of the total contaminant output during remediation period, leaching of NAPL was 19 % lower and that of dissolved contaminants 7–11 % lower in the biostimulation treatment than in the natural attenuation treatment. In contrast, the chemical oxidation mobilized contaminants and NAPL emissions were 15 % and dissolved contaminants 8–18 % higher than in the natural attenuation. The oil-leaching process can be divided into the following four stages: distribution, adsorption, desorption, and leaching. Oil contaminants exist in soil as the following four different phases: as NAPL, adsorbed to soil particles, dissolved in water or in a gaseous form, and can transform between these under changing conditions (Yang et al. 2013). In the phenomenon called aging, the interactions between contaminants and soil become stronger and decrease contaminant bioavailability (Hatzinger and Alexander 1995). In freshly contaminated soils, as studied here, strong binding has not been evolved yet and a large part of contaminants was in a mobile phase (NAPL or dissolved in water). Our results show that the enhanced bacterial degradation via biostimulation decreased the oil contaminants in the mobile phase and significantly reduced the total oil migration through the soil. This is a remarkable achievement for a biostimulation treatment carried out in situ, and especially valuable in groundwater areas, where the most significant risk is the leakage of contaminants into the groundwater.

When contaminant biodegradation in soil is evaluated using small-scale laboratory experiments, contaminant degradability may be properly demonstrated, but degradation rate is usually overestimated due to overly favorable conditions (Aichberger et al. 2005). In large-scale experiments, soil spatial heterogeneity and varying environmental conditions give a more realistic prediction of the remediation performance in a real case. However, besides the higher experimental costs, the challenge in large-scale experiments is to build uniform, replicated soil columns. For instance, although we aimed at constructing comparable soil columns, leaching times and water and NAPL volumes differed among replicate lysimeters even before the remediation treatments were started. Soil contaminant and bacterial abundances also deviated among replicate samples taken with the auger throughout the experiment, and the average SDs were 17–73 % higher than in the end composite samples that better represented the different soil layers. This highlights the importance of comprehensive sampling also in real cases; high number of replicate samples increases the reliability of results, and consequently, the estimation of the state of the contaminated area is more accurate.

**Fig. 7:**
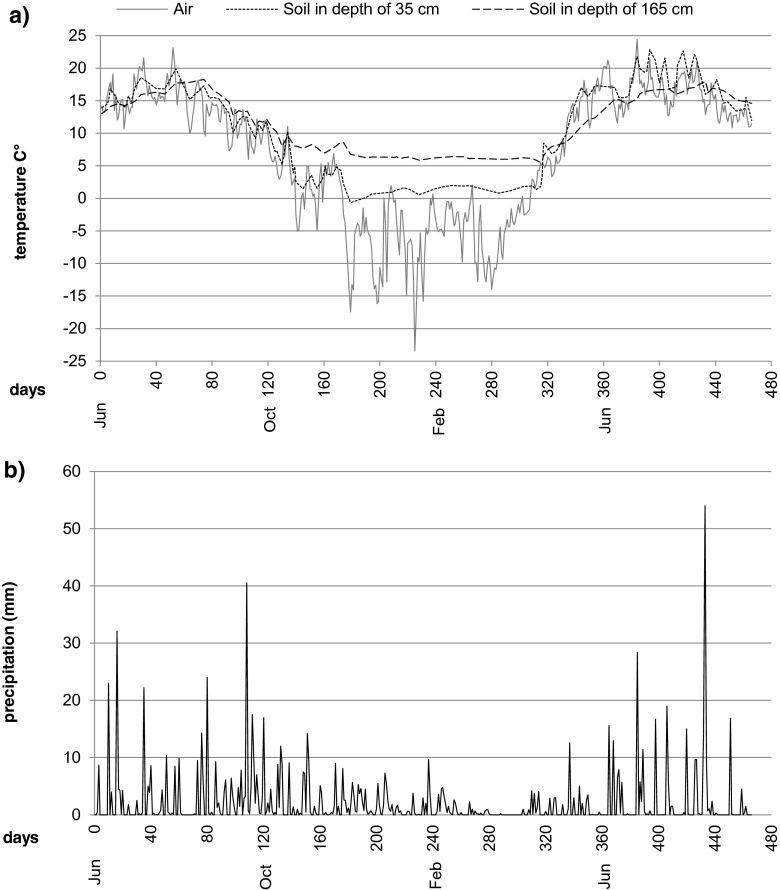
Weather conditions during the experiment. In **a**, air temperature is shown as daily average and soil temperature as the average of eight lysimeters. In **b**, daily precipitation includes the snowfall which has been transformed to rainfall. Air temperature and precipitation data are provided by the Finnish Meteorological Institute from the Laune meteorological station, located around 3 km from the experiment area

Soil temperature in lysimeters was beneath 10 °C nearly half of the experiment duration (Fig. 7). This represents well the conditions in Finland, where the mean annual temperature beneath the subsurface soil remains below 10 °C (Yli-Halla and Mokma 1998). In low temperatures, biodegradation is slow due to low microbial activity and high oil viscosity and insolubility, which decrease oil bioavailability (Margesin 2000). However, it does not cease totally even on winter time, as observed also in this study. Decreased contaminant amounts both in soil and in water after winter proved that bioremediation process continued also during the cold period (Figs. 2 and 3). Another factor delaying degradation in our study was probably soil dryness in summer months (soil moisture content 3–5 % of fresh mass), but this also corresponds with the real life as the soil type and infiltration rate were the same as in the Iisalmi case. In fact, the C_10_–C_40_ hydrocarbon removal via biostimulation in Iisalmi was at a similar level as in our experiment when equal treatment duration at the same season was compared (Final report 13.4.2016, Eteläntie, Iisalmi, after-treatment operations in tank truck accident, Ramboll Finland Ltd., not in public use). Using a real accident case as a model for the implementation of this experiment demonstrated how an experimental study can give useful information about processes taking place in the field. Our pilot-scale experiment simulated the natural conditions and expressed realistic remediation rate and also revealed the challenges of different treatments and factors that can influence the remediation process.

## Conclusions

Our study proved that a large pilot-scale experimental simulation can generate realistic predictions about remediation performance in the field. Soil heterogeneity was clearly observable in this scale and highlighted the importance of comprehensive sampling in real cases. The changes in soil environmental conditions induced by the treatments were visible as changes in soil microbial community structure, suggesting that community fingerprinting could be used as an indicator of the soil microbial responses in bioremediation treatments. In a fresh oil contamination accident, as studied in this experiment, microbes seem to be efficient in degrading the easily available oil, dissolved in the water phase, and biostimulation can lead to a reduced amount of contaminant leachate from the soil. Biostimulation can also enhance soil purification as shown by our more comprehensive, destructive end sampling. These findings suggest that the biostimulation treatment could be used not only for soil cleaning but also for reducing the leaching of oil through the soil column and preventing groundwater contamination. In contrast, the chemical oxidation, carried out using realistic peroxide volumes possible also in real cleanup cases, did not speed up soil cleaning and enhanced the mobilization of contaminants. We conclude that doing nothing (i.e., using natural attenuation) is worse for the environment than employing an efficient action such as biostimulation, but on the other hand, a wrong choice of actions, as exemplified by our chemical oxidation treatment, may also worsen the situation.

## Electronic supplementary material

ESM 1 (PDF 86 kb)

## Abbreviations

NAPL: Non-aqueous phase liquid
BTEX: Benzene, toluene, ethylbenzene, and xylene
MTBE: Methyl tertiary butyl ether
TAME: Tertiary amyl methyl ether
TAEE: Tertiary amyl ethyl ether
ETBE: Ethyl tertiary butyl ether
ISCO: In situ chemical oxidation
VOCs: Volatile organic compounds
