# Supplementary material for: Biostimulation proved to be the most efficient method in the comparison of in situ soil remediation treatments after a simulated oil spill accident
Source: Environ Sci Pollut Res Int. 2016 Sep 27;23(24):25024–38. doi: 10.1007/s11356-016-7606-0 (PMC5124059; doi:10.1007/s11356-016-7606-0)
Supplement: Supplementary file 1 — (PDF 86 kb) [file 11356_2016_7606_MOESM1_ESM.pdf]

Supplementary data

Title: Biostimulation proved to be the most efficient method in the comparison of in situ soil remediation treatments after a simulated oil spill accident

Journal name: Environmental Science and Pollution Research

Authors: Suvi Simpanen[a], Mari Dahl[a], Magdalena Gerlach[a], Anu Mikkonen[b], Vuokko Malk[ac], Juha Mikola[a] and Martin Romantschuk[a]

[a]University of Helsinki, Department of Environmental Sciences, Niemenkatu 73, 15140 Lahti, Finland
[b]University of Jyväskylä, Department of Biological and Environmental Science, Survontie 9 C, 40014, Jyväskylä, Finland
[c]Mikkeli University of Applied Sciences, Patteristonkatu 3, 50100 Mikkeli, Finland

Corresponding author: Suvi Simpanen, e-mail: suvi.simpanen@helsinki.fi

Table S1 The contaminant quantities that migrated into the water before the start of remediation treatments calculated as percentages of the total leachate quantities (mean ± SD).

Contaminant	Natural attenuation	Chemical oxidation	Biostimulation
NAPL	61 ± 6	46 ± 22	80 ± 8
C_{10}-C_{40} hydrocarbons	84 ± 5	66 ± 3	91 ± 1
C_5-C_{10} hydrocarbons	52 ± 4	44 ± 8	63 ± 4
BTEX-compounds	57 ± 3	42 ± 6	65 ± 3
TAEE	95 ± 2	80 ± 21	90 ± 7
ETBE	100 ± 0	100 ± 0	100 ± 0
